# [Retracted] Characterization of sonic hedgehog inhibition in gastric carcinoma cells

**DOI:** 10.3892/ol.2026.15850

**Published:** 2026-09-08

**Authors:** Ruxue Bai, Hongchuan Zhao, Xiang Zhang, Shiyu Du

Oncol Lett 7: 1381–1384, 2014; DOI: 10.3892/ol.2014.1964

Following the publication of the above paper, it was drawn to the Editor's attention by a concerned reader that the Transwell assay images shown in Fig. 2A on p. 1383 subsequently appeared in another paper written by different authors that was also published in *Oncology Letters* [Gu H, Li X, Zhou C, Wen Y, Shen Y, Zhou L and Li J: Effects and mechanisms of blocking the hedgehog signaling pathway in human gastric cancer cells. Oncol Lett 9: 1997–2002, 2018], although larger fields of vision of the images were presented in that paper, suggesting that that paper may have been in possession of the original images. In addition, a concern was raised by a different commentator that a pair of the bands for the Gli1 data in Fig. 3 appeared to be non-contiguous with the remainder of the bands in the gel, suggesting that this figure may have been inappropriately edited.

Given that these issues have come to light, the Editor of *Oncology Letters* has decided that this paper should be retracted from the Journal on account of a lack of confidence in the presented data. The authors were asked for an explanation to account for these concerns, but the Editorial Office did not receive a reply. The Editor apologizes to the readership for any inconvenience caused.

